# Daily injection of melatonin inhibits insulin resistance induced by chronic mealtime shift

**DOI:** 10.14814/phy2.15227

**Published:** 2022-03-28

**Authors:** Jihyun Park, Jichul Kim, Yejin Yun, Dong‐Hee Han, Kyungjin Kim, Jongki Hong, Sehyung Cho

**Affiliations:** ^1^ Department of Neuroscience Graduate School Kyung Hee University Seoul South Korea; ^2^ Department of Life & Nanopharmaceutical Science Graduate School Kyung Hee University Seoul South Korea; ^3^ Department of Biomedical Science Graduate School Kyung Hee University Seoul South Korea; ^4^ Department of Brain Science DGIST Daegu South Korea; ^5^ College of Pharmacy Kyung Hee University Seoul South Korea; ^6^ Department of Physiology Kyung Hee University School of Medicine Seoul South Korea; ^7^ Present address: Ildong Pharmaceutical Co. Ltd Seocho‐gu Seoul Republic of Korea

**Keywords:** insulin resistance, mealtime shift, melatonin, shift work, type2 diabetes

## Abstract

Shift work disorders have become an emerging concern worldwide. Shift disorders encompass a wide range of illnesses that have yet to be identified. The study focused on the relationship between shift work disorders and insulin resistance. Previously, it was reported that advancing the usual mealtime of mice triggered insulin resistance. Here, the hypothesis that chronic mealtime shifts induce oxidative damage leading to chronic diseases such as type 2 diabetes was tested. It was found that mealtime shift causes imbalances between anti‐oxidative capacity and reactive oxygen species (ROS) levels, indicating increased oxidative damage during the light/rest phase. This study further demonstrated that daily supplementation of antioxidants at the appropriate time of day inhibited insulin resistance caused by chronic mealtime shifts, suggesting significant and chronic health implications for shift workers. In conclusion, it was confirmed that increased ROS levels caused by mealtime shift induce insulin resistance, which is inhibited by the antioxidant melatonin.

## Graphical abstract

We focused on the relationship between Shift work disorders and insulin resistance. We further demonstrate that daily supplementation of antioxidants at the appropriate time of day inhibits insulin resistance caused by chronic mealtime shift, suggesting significant and chronic health implications for shift workers.

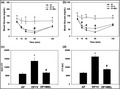

## 1 INTRODUCTION

Nearly all organisms on the planet Earth exhibit behavioral, physiological, and biochemical rhythms with a 24‐h periodicity. Distinct intrinsic molecular oscillators are believed to coordinate diverse biological processes under circadian rhythms (Reinke & Asher, 2019). The molecular and behavioral rhythms driven by the circadian oscillators enable organisms to anticipate and benefit from daily environmental changes. Thereby, circadian rhythms affect the organism's survival and fitness via appropriate behavioral adaptation on a daily basis (Brown, 2016; Reinke & Asher, 2019; Zimmet et al., 2019). The significance of these endogenous circadian rhythms is best appreciated under severe alteration, either via pathological changes due to genetic defects within the core clock genes or by chronic circadian disturbances *per se* (Brown, 2016; Noh et al., 2011; Park et al., 2012; Reinke & Asher, 2019; Yoon et al., 2012; Zimmet et al., 2019). Humans are active predominantly during the light phase. With the advent of light bulbs, however, individuals increasingly tend to work at unusual times of the day in most industrialized countries. For example, a significant proportion (16~28%) of the population works in various shifts currently (Kervezee et al., 2018; Kolbe‐Alexander et al., 2019). Several lines of epidemiological evidence clearly indicate that those who work in shifts are more likely to develop chronic illnesses, such as obesity, type 2 diabetes, cardiometabolic consequences, and sleep disturbances (Lim et al., 2018; Liu et al., 2018; Zimmet et al., 2019; Zoto et al., 2019). Thus, the precise mechanisms linking circadian disturbances to chronic diseases and the possible strategies to prevent them need to be identified. To address these issues, appropriate animal models of circadian disturbance are required. Specifically, as the feeding/fasting cycle plays an important role in metabolic homeostasis, it has been speculated that having meals at unusual times of day may lead to disturbances in metabolic rhythm (Yoon et al., 2012).

Gehring and Rosbash (2003) once hypothesized that circadian rhythms or the intrinsic molecular oscillators had evolved to minimize UV‐induced damage during the early metazoan period. As UV irradiation increases reactive oxygen species (ROS) levels and induces apoptosis of the affected cells (Makrantonaki & Zouboulis, 2007), it is tempting to speculate that circadian disturbances may dysregulate ROS levels and lead to subsequent oxidative stress‐related disorders. In support of this notion, almost all shift work disorders (Shift work disorders) mentioned above are closely related to increased oxidative stress (Teixeira et al., 2019). Moreover, genetic defects within the core clock genes have been implicated in the dysregulation of oxidative stress involving the brain and the peripheral tissues (Lee et al., 2013; Musiek et al., 2013).

Oxidative stress is related to Shift work disorders (Gibson., 2022; Teixeira et al., 2019). In fact, it is known that oxidative stress is related to insulin resistance, which is one of the most important features of type 2 diabetes (Demir et al., 2016). Although the pathway linking oxidative stress and insulin resistance is not well‐known, active investigations are ongoing. In this study, we resolved the link between oxidative stress and type 2 diabetes, one of the Shift work disorders.

Previously, we reported that feeding mice chronically at unusual times during the day perturbs the circadian rhythm along with behavioral and metabolic alterations including insulin resistance (Yoon et al., 2012). Thus, in the present study using the same animal model of mealtime shift, we first investigated the pattern of circadian activity in the mice. We then attempted to evaluate the hypothesis of dysregulation of ROS levels by chronic circadian disturbances resulting in oxidative stress‐related disorders like insulin resistance. To this end, we first examined how mealtime shift affects blood ROS levels and anti‐oxidative capacity throughout a circadian cycle. We then investigated the effects of strong antioxidants such as melatonin on blood ROS levels and antioxidative capacity. Finally, we tested whether daily injection of antioxidants can prevent mealtime shift‐induced insulin resistance and change metabolic factors.

## 2 MATERIALS AND METHODS

### 2.1 Materials

Melatonin was purchased from Sigma (St. Louis, MO, USA). Melatonin was first dissolved in absolute ethanol and diluted 50‐fold in physiological saline prior to injection. Free oxygen radical defense test (FORD) and free oxygen radical test (FORT) assay kits were obtained from Callegari (Parma, Italy). Other chemicals and reagents, unless mentioned otherwise, were obtained from Sigma.

### 2.2 Animals care and handling

All animal experiments were approved by the Kyung Hee University Institutional Animal Care and Use Committee (Permit number: KHUASP(SE)‐11‐035) and performed under its guidelines. All the animals were treated to minimize suffering. C57BL/6J male mice (7 weeks old) were purchased from DBL (Seoul, Korea). Upon arrival, mice were acclimatized to a temperature‐controlled room (23 ± 1°C) with a 12:12‐light–dark (LD) photoperiodic cycle with food and water available all the time. Under experimental settings, mice were housed in a light‐proof clean animal rack cabinet (Shin Biotech, Seoul, Korea) with light intensity during the light phase maintained at 350~450 lux at the bottom of the cage. Mice were continuously fed with normal food chows *ad libitum* until the initiation of scheduled feeding.

### 2.3 Restrictive feeding schedule

Mice were randomly divided into three groups. The first group of mice (AF; *ad libitum* group) was continuously fed *ad libitum* during the experimental period. The other two groups of mice were fed only during the late day (DF; daytime feeding groups, ZT06~ZT11) with daily intraperitoneal (*i*.*p*.) injection of either vehicle control (2% ethanol in physiological saline) or melatonin (Sigma, M5250) as an antioxidant. Melatonin was used at 10 mg/kg in mouse body weight.

### 2.4 Determination of oxidants and antioxidants levels in the blood

Reactive oxygen species (ROS) levels were determined using the FORT. In this test, the extent of free radicals produced by the reaction was directly proportional to the number of lipid peroxides present in the blood sample (25 μl), which reacted with a phenylenediamine derivative that forms a radical molecule. This molecule was detected by a spectrophotometer at 505 nm (Form CR3000, Callegari, Parma, Italy). Results were expressed in FORT units, where 1 FORT unit corresponds to 0.26 mg/L of H_2_O_2_. Antioxidant capacity was evaluated by a Trolox equivalent antioxidant capacity assay using the FORD. In the presence of an acidic buffer (pH 5.2) and a suitable oxidant (FeCl_3_), the chromogen containing 4‐amino‐*N*,*N*‐diethylaniline sulfate formed a stable and colored radical cation that was photometrically detected at 505 nm. Antioxidant compounds in the blood sample (45 μl) reduced the radical cation of the chromogen, resulting in decoloration of the solution, which was proportional to the antioxidant concentrations.

### 2.5 Activity monitoring

To measure the locomotor activity and body temperature, mice were exposed to light ether anesthesia and surgically implanted with a G2 E‐mitter probe (Mini Mitter, Oregon, USA.) on the dorsal neck under the skin (Makrantonaki & Zouboulis, 2007). Body temperature (BT) and home‐cage activity (HCA) from E‐mitter probe were registered by a receiver placed under the cage and connected to the computer. Wheel running activity (WRA) was measured by the switch and magnetic bar placed laterally on the running wheel (12 cm in diameter and 5.4 cm wide) connected to the computer. BT, HCA, and WRA were measured using the Activity Monitoring System (Mini Mitter, Oregon, USA) and recorded at 6‐min intervals using the VitalView® Data Acquisition System. Individual actograms were obtained using the ActiView® software. To obtain the daily pattern, monitoring results retrieved as a Microsoft® Excel file for the whole recording period were either averaged (in case of BT) or summed up (in case of HCA and WRA) as one‐hour long bins and the resulting 8 weeks profiles were pooled according to the indicated ZT.

### 2.6 Glucose tolerance test and insulin tolerance test

Previously, we observed that the time of the day had minimal impact on the results of the glucose tolerance test (GTT) and insulin tolerance test (ITT) (Yoon et al., 2012). Thus, we performed GTT and ITT after 16 h fasting since the last scheduled meal. For GTT, D‐glucose (Sigma, G5767; 2 g/kg body weight) was injected intraperitoneally (ip). For ITT, insulin (Lilly, HI0210, 0.5 U/kg body weight) was injected *i*.*p*. after 16 h fasting. Blood samples were collected at 0, 15, 30, 60, and 120 min for the determination of blood glucose levels as previously described (Yoon et al., 2012).

### 2.7 Statistical Analysis

Data were expressed as the mean ± SEM and analyzed using the one‐way ANOVA. Student's *t*‐test was used for statistical comparisons between the two groups. Statistical significance was set at *p* < 0.05.

## 3 RESULTS

### 3.1 Chronic mealtime shift significantly affects antioxidative capacity and ROS levels differently within the blood

**FIGURE 1:**
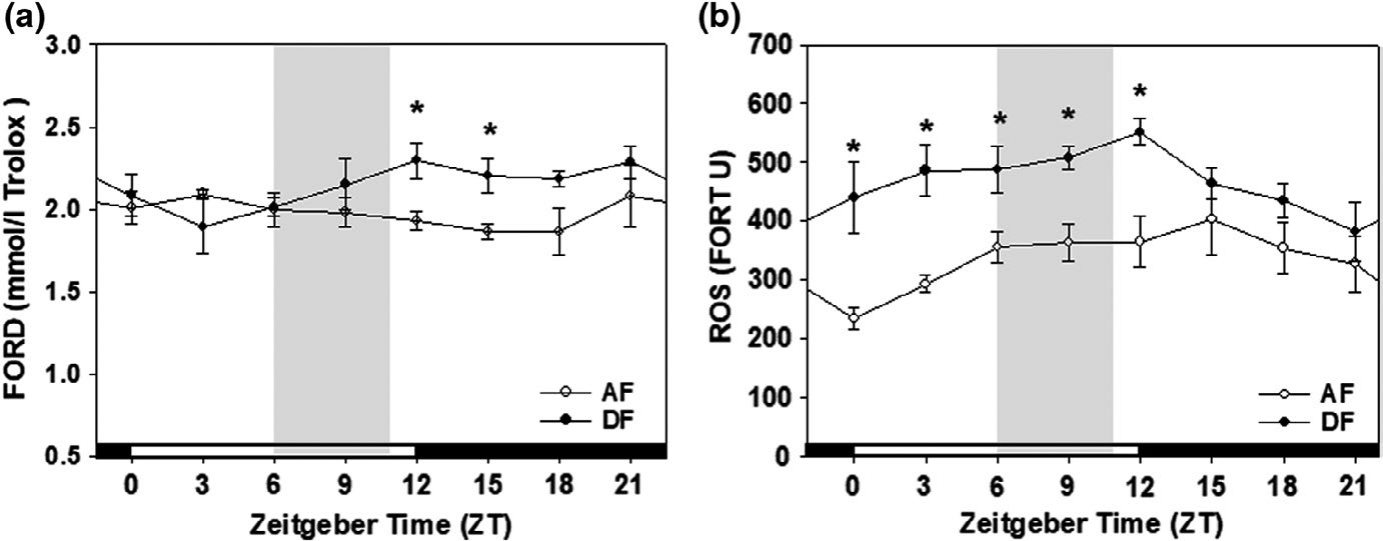
Antioxidant capacity and ROS level in AF and DF group. The effects of mealtime shift on anti‐oxidative capacity (a) and ROS levels (b) within the blood vary across a circadian cycle. Young adult male mice were first entrained to a 12:12 LD photoperiodic cycle for a week with food and water provided *ad libitum*. The mice were fed either *ad libitum* (AF, open circle) or in a time‐restrictive manner during the late daytime (DF, closed circle) for 4 weeks. Blood anti‐oxidative capacity (a) and ROS levels (b) were determined at 3‐h intervals throughout a circadian cycle using FORD and FORT assay kits, respectively. Shades indicate the duration of time‐restrictive feeding in DF group. All data are expressed as mean ± SEM. (*n* = 3–6 per each time point). *p < 0.05 versus AF group

First, we analyzed the diurnal variations of anti‐oxidative capacity and ROS levels within the blood of mice fed *ad libitum*. Blood antioxidative capacity as determined by the FORD assay showed minimal variation under a 12:12 LD photoperiodic cycle (Figure 1a, open circles). Nevertheless, it is plausible that the antioxidative capacity is relatively high during the light/rest phase and low during the dark/active phase in this nocturnal animal. In contrast, blood ROS levels measured using the FORT assay showed a pronounced diurnal variation under the study design adopted. An almost twofold difference between nadir (at ZT0) and zenith (around ZT12) was clearly visible (Figure 1b, open circle). Thus, we conclude that both antioxidative capacity and ROS levels display distinct diurnal variations in mice fed *ad libitum*. Next, we investigated the effects of chronic daytime feeding (DF; Yoon et al., 2012) on antioxidative capacity and ROS levels after 4 weeks of scheduled feeding. As shown in Figure 1 (closed circles), both were significantly affected, but in a different manner. Significant increases in anti‐oxidative capacity during the early scotophase and large increases in ROS levels during the light phase were observed prominently. Therefore, we conclude that feeding mice during the late light/rest phase significantly disrupt homeostatic regulation of the antioxidative defense system. Moreover, we can expect that anti‐oxidative defense will be severely hampered during the light phase in this animal model.

### 3.2 Daily injection of antioxidants increases antioxidative capacity and blocks mealtime shift‐induced increases in ROS levels

**FIGURE 2:**
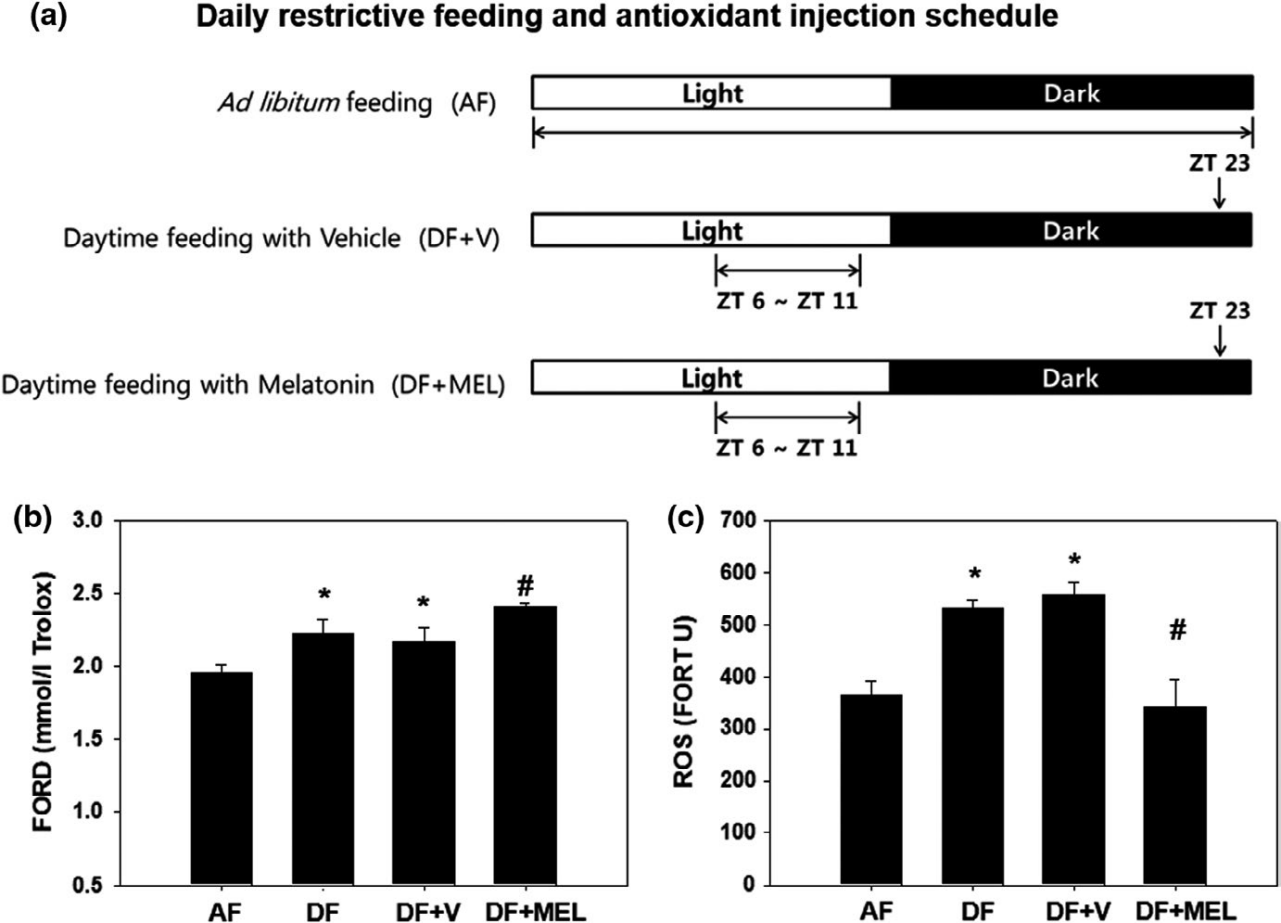
Experimental scheme and FORD/FORT assay result in AF, DF, DF + V, and DF + MEL. Young adult C57BL/6J male mice (8 weeks old) were first entrained to a 12:12 LD cycle for 1 week with food and water *ad libitum*. (a) The mice were randomly divided into three groups: Food available *ad libitum* (AF), food available during the late day (ZT 6–11) with daily *i*.*p*. injection of vehicle (2% ethanol in physiological saline) at ZT 23 (DF + V) and food available during the late day with daily *i*.*p*. injection of melatonin (10 mg/kg body weight) at ZT 23 (DF + MEL). Drug administration was conducted under dim red light. Water was available all the time in all groups. (b), (c) Daily injection of antioxidants increases the antioxidative capacity and blocks mealtime shift‐induced increases of ROS levels. Mice were fed either *ad libitum* (AF) or in a time‐restricted manner during the late daytime (DF) for 6 weeks as shown in (a). Sets of DF mice were injected *i*.*p*. with a vehicle or melatonin daily at ZT23 for the last 2 weeks of scheduled feeding. FORD/FORT assays were performed between ZT9 and ZT12. All data are expressed as mean ± SEM (*n* = 10–13 per group). *p < 0.05 versus AF group. #*p* < 0.05 versus DF group

In our animal model of mealtime shift, increased oxidative damage was expected during the light phase. Thus, we decided to inject antioxidants daily at ZT23 just before any increases in ROS levels ensue. Melatonin (MEL) was selected as the antioxidant due to its strong antioxidative activity and is widely used in various oxidative stress‐related studies (Noh et al., 2014; Yu & Tan, 2019). Mice were fed either *ad libitum* (AF) or during the late daytime (DF) for 6 weeks. For the last two weeks of scheduled feeding, MEL (10 mg/kg body weight) was injected *i*.*p*. every day at ZT23 (Figure 2a). The antioxidative capacity and ROS levels within the blood were determined between ZT9 and ZT12. As shown in Figure 2b, the mealtime shift (DF group) slightly but significantly increased the antioxidative capacity at this time point (see also Figure 1a). More importantly, daily injection of MEL further increased the antioxidative capacity in these animals. Moreover, MEL efficiently blocked the mealtime shift‐induced increase in ROS levels (Figure 2c). Thus, daily single injections of strong antioxidants like MEL at appropriate times during the day may be used to block the oxidative damage induced by mealtime shifts.

### 3.3 Time‐restrictive feeding considerably alters WRA, BT, and HCA rhythms in young adult male mice

To investigate the possible changes of body temperature and behavioral rhythms caused by mealtime shift, young adult male mice, surgically implanted with E‐mitter probes, were fed time‐restrictedly as shown schematically in Figure 2

**FIGURE 3:**
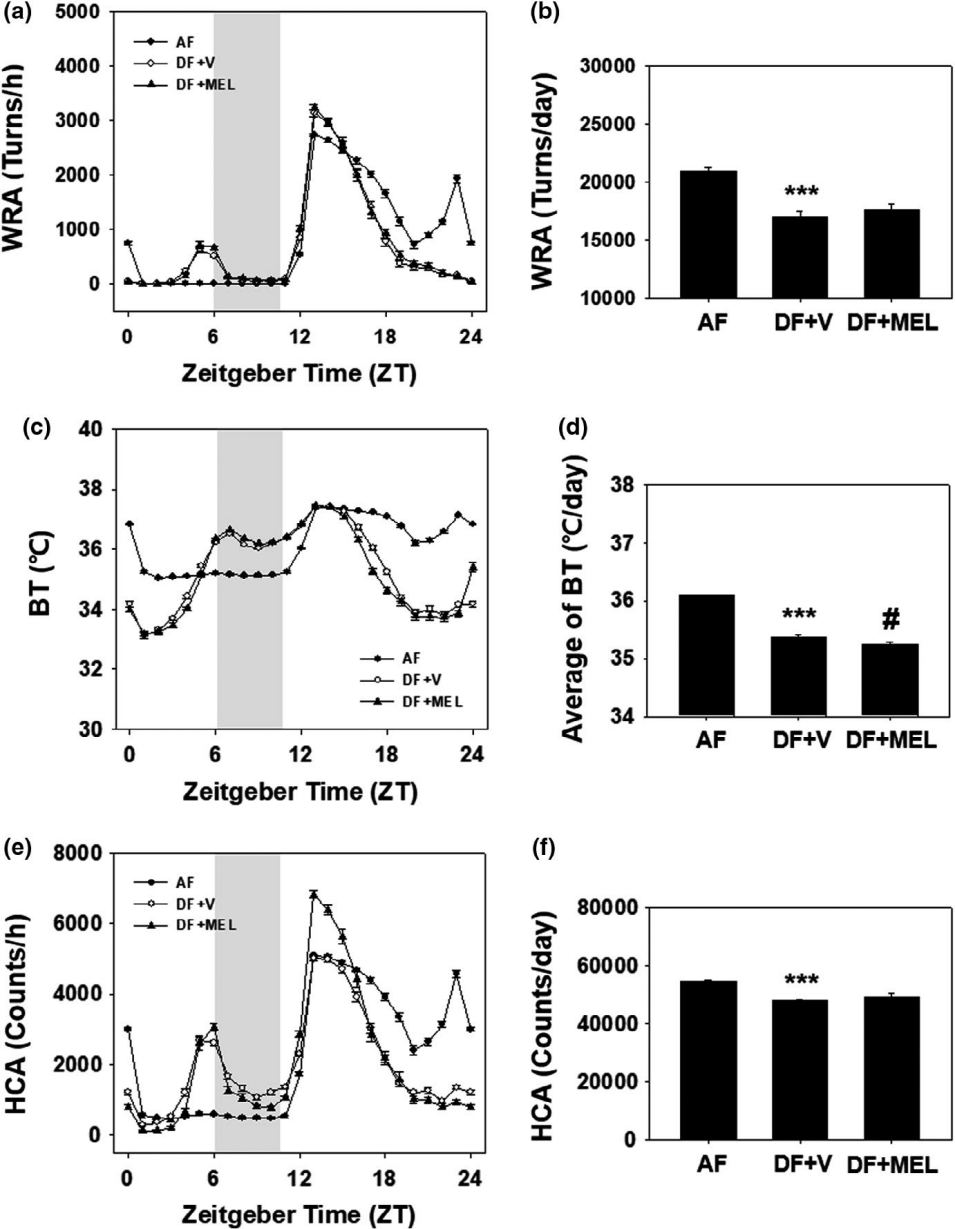
Daily rhythms of WRA, BT, and HCA of young adult male mice under time‐restrictive feeding. Young adult male mice (8 weeks old), surgically implanted with E‐mitter probes, were first entrained to a 12:12 LD photoperiodic cycle for 2 weeks with food and water provided *ad libitum*. Mice were then exposed to time‐restricted feeding as schematized in Figure 2A. Daily patterns of WRA (a), BT (c) and HCA in AF, DF + V, and DF + MEL (e) are shown. Gray bars indicate meal times in day‐time feeding. To obtain the daily patterns, monitoring results for the whole time‐restricted period were either summed up (in case of WRA and HCA) or averaged (in case of BT) as 1 h bin and the resulting 8 week profiles were pooled according to the indicated ZT (mean ± SEM, *n* = 4 per group). ***p < 0.005 versus AF; #*p* < 0.05 versus DF + V

A. During the 2 weeks of entraining and following 8 weeks of time‐restricted feeding, WRA, BT, and HCA were continuously recorded. Average daily profiles for the 8 weeks of time‐restricted feeding are depicted in Figure 3. Compared with AF, DF mice showed increased WRA from ZT4 until the mealtime, which is a well‐recognized food‐anticipatory activity in mice exposed to time‐restricted feeding (Figure 3a). Time‐restricted feeding induced a rapid decline in body temperature during the night and increase in body temperature during the mealtime (Figure 3c). Changes in HCA rhythms are comparable to those of WRA (Figure 3e). Daily supplementation of MEL did not significantly alter the WRA and HCA rhythms compared with the DF group (Figure 3b and f). Melatonin supplementation significantly increased only the HCA rhythm during the early night (Figure 3E). As shown in Figure 3b, d, and f, daytime feeding reduced daily WRA and HCA as well as the averaged body temperature, which was not significantly altered by daily MEL supplementation.

### 3.4 Daily injection of antioxidants prevents insulin resistance induced by chronic mealtime shift

**FIGURE 4:**
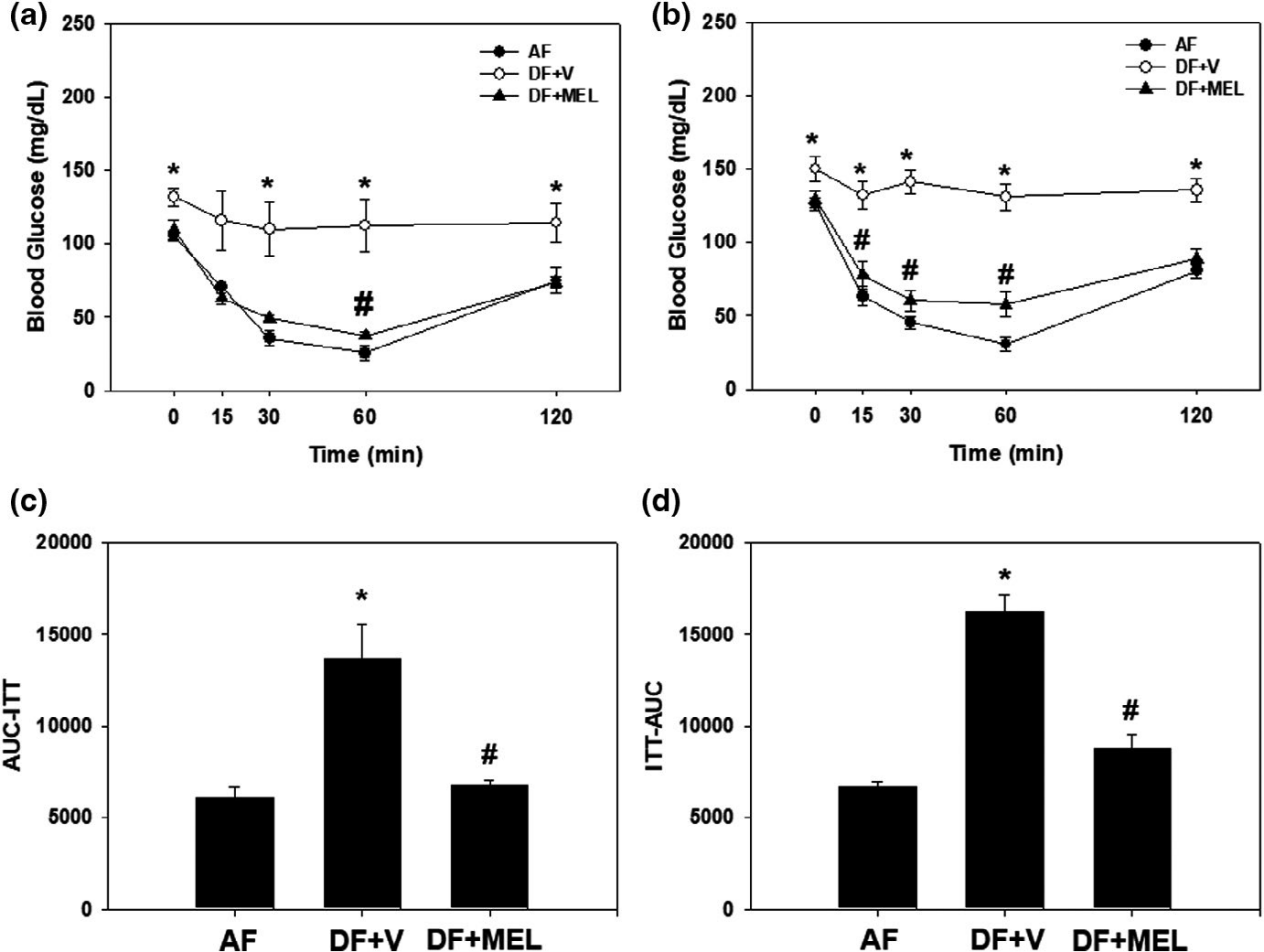
Daily injection of antioxidants prevents mealtime shift‐induced insulin resistance. Young adult male mice were first entrained to a 12:12 LD photoperiodic cycle for a week and then fed either *ad libitum* or in a time‐restricted manner for 13 consecutive weeks with a daily injection of vehicle or melatonin as shown in Figure 2A. Insulin tolerance tests were performed at Weeks 11 (a and c) or 13 (b and d) in the scheduled feeding. Time‐course changes of blood glucose levels in response to insulin (a and b) and area under the curve (c and d) are shown here. All data are expressed as mean ± SEM (*n* = 6 per group). *p < 0.05 versus AF group; #*p* < 0.05 versus vehicle‐injected group

We examined the effect of daily injection of MEL on insulin resistance induced by chronic mealtime shifts. After a week of acclimatization, mice were fed either *ad libitum* (AF) or during the late daytime (DF) with or without daily injection of antioxidants at ZT23 for 13 consecutive weeks. GTTs or ITTs were performed at the 9th, 11th, or 13th weeks of scheduled feeding. GTT performed at week 9 did not reveal any significant difference among the groups (Figure S3). However, ITT performed at week 11 (Figure 4a and c) or 13 (Figure 4b and d) revealed dramatic effects of MEL. The chronic mealtime shift definitely induced insulin resistance (see DF + V group in Figure 4), reproducing previous observations (Yoon et al., 2012). More importantly, daily injection of antioxidants at ZT23 ameliorated insulin resistance induced by chronic mealtime shifts. Overall, the mealtime shift negatively affected the antioxidative defense system leading to metabolic disturbances like insulin resistance, which can be efficiently blocked by daily supplementation of antioxidants at the appropriate time of the day.

## 4 DISCUSSION

In modern society, shift workers with frequent disruption in their circadian rhythms, are known to be increasingly vulnerable to chronic illnesses such as cancer, obesity, type 2 diabetes, hypertension, cardiovascular diseases, gastrointestinal problems, urological problems, irregular menstruation, infertility, accelerated aging, premature deaths, and various psychiatric/mental problems (Jin et al., 2017; Kervezee et al., 2018; Lim et al., 2018; Liu et al., 2018; Noh et al., 2011; Park et al., 2012; Yoon et al., 2012; Zimmet et al., 2019; Zoto et al., 2019). Intrinsic circadian oscillators are estimated to have first appeared in the history of life about 2.3 billion years ago under the great oxidative event (Karafyllidis, 2012). Their evolution as a defense mechanism to prevent excessive stress (Gehring & Rosbash, 2003) and chronic disturbances of circadian rhythms disrupted the homeostatic regulation of stress management, resulting in the prevalence of stress‐related disorders. In the current study, we showed that feeding mice at unusual times of the day leads to an imbalance between antioxidative capacity and ROS levels, indicating increased oxidative damage during the light/rest phase. Further, we demonstrated that daily supplementation of melatonin prevents mealtime shift‐induced insulin resistance, a major symptom of type 2 diabetes.

In the present work, we utilized the same animal model of mealtime shift reported previously (Yoon et al., 2012). where animals were fed in a time‐restricted manner to advance their usual mealtime by 6 h. The chronic mealtime shift not only alters circadian rhythms and behaviors but leads to disturbances in various metabolic rhythms, resulting in severe insulin resistance. In this study, we not only reproduced the previous findings but also showed that antioxidants supplied daily at the proper time of day prevent insulin resistance, suggesting that this animal model represents a more realistic experimental model of type 2 diabetes than others currently used (Al‐Awar et al., 2016; Bowe et al., 2014; Bunner et al., 2014; Skovsø, 2014; Yin et al., 2019).

When fed freely, nocturnal mice consume most of their food in the early scotophase (Sanchez‐Alavez et al., 2017; Yoon et al., 2012). Under this condition, blood antioxidative capacity remains almost constant or displays a diurnal rhythm with a tiny amplitude, while blood ROS level exhibits a prominent rhythm with a large amplitude (Figure 1). ROS level reaches the nadir at the end of scotophase, gradually increases during the light/rest phase, and reaches the zenith around the beginning of the dark phase. Such a prominent rhythm reflects the expression of diverse molecular regulators including anti‐ and pro‐oxidative enzymes within the body (Cao et al., 2015; Xu et al., 2012), which requires further investigation.

Advancing the usual mealtime of mice by 6 h led to diurnal variations in antioxidative capacity and ROS levels (Figure 1). The antioxidative capacity significantly increased in the early night while the levels of ROS showed huge increments during the light phase. These changes result in imbalance between oxidative stress and antioxidative activity, indicating increased oxidative damage during the light phase. Therefore, we administered melatonin, a strong antioxidant (Skovsø et al., 2015; Yu & Tan, 2019), at ZT23 just before any damage was expected. As a result, the blood antioxidative capacity was significantly increased, and mealtime shift‐induced increases of ROS levels were almost completely blocked by these treatments (Figure 2), suggesting that administering an adequate amount of antioxidants at the appropriate time of day can prevent oxidative damage adequately. Nevertheless, further studies are needed to investigate and identify the most efficient antioxidants, proper dosages, and their feasibility in human application, especially in shift workers.

We determined whether antioxidants affected locomotor activity and body temperature rhythms induced by time‐restricted feeding (Figures S1 and S2). Time‐restricted feeding caused a strong torpor‐like symptom, which was not prevented by the antioxidant treatment. Torpor refers to a state of reduced physiological activity in small animals, a response to increased survival under deficient nutritional intake. Administration of melatonin further lowered body temperature than vehicle treatment (Figure 3d), suggesting that melatonin increased the incidence of daily torpor. Effects of melatonin on HCA and locomotor activity were not significantly different from those of vehicles (Figure 3b and f). Administration of melatonin is known to reduce locomotor activity (Musiek et al., 2013). Accordingly, torpor induced by time‐restricted feeding appears to be enhanced by melatonin at some time points (Figure 3e). However, the amplitude of activity in ZT12‐16 significantly increased under time‐restricted feeding in this experiment and may improve health status.

Most of the shift work disorders are closely related to diseases caused by increased oxidative stress. According to a recent study, there is an increase in oxidative stress indicators in night workers, such as increased DNA damage, reduced DNA recovery ability, and reduced antioxidant defense (Gibson., 2022). However, to infer a causal relationship between shift work and increased oxidative stress, additional studies are needed to confirm the exact mechanism. In this study, oxidative stress increased due to damaged circadian rhythm caused by mealtime shifts, resulting in insulin resistance.

It was discovered that when mice with insulin resistance were given melatonin, a powerful antioxidant, as a mealtime shift, their insulin levels returned to normal. Although it was not possible to reproduce the broken circadian rhythm that occurs in shift workers, considering that food intake by shift workers occurs mainly at night time, the rest phase (Lennernäs et al., 1995), mealtime shift could be one of the causes of the broken circadian rhythm of shift workers. Therefore, it was possible to find out that high oxidative stress in shift workers was correlated with increased oxidative stress due to mealtime shifts. In addition, when melatonin, a powerful antioxidant, was administered, the insulin level was restored to normal along with the reduction of oxidative stress, suggesting a relationship between the oxidative stress mechanism and circadian rhythm.

The relationship between the increase in oxidative stress and insulin resistance requires further mechanism studies. Based on these studies, it can be interesting to assess how antioxidants lower insulin levels. It is necessary to evaluate the effects of antioxidants on possible prevention and treatment of other chronic diseases and to elucidate the precise mechanisms. More importantly, the feasibility of antioxidants should be investigated in a wide range of clinical trials in human subjects, especially in shift workers.

In conclusion, it can be said that mealtime shift affects diurnal rhythms in antioxidative capacity and ROS levels differently. Accordingly, supplementation of antioxidants during the day at specific times not only prevents increases in ROS levels but also overcome insulin resistance.

## CONFLICT OF INTEREST

The author does not declare conflicts of interest that may be perceived as impeding the fairness of the reported study.

## AUTHOR CONTRIBUTION

Conceived and designed the experiments: JP, DHH, SC. Performed the experiments: JP, JK, YY, DHH, JH, SC. Analyzed the data: JP, JK, YY, DHH, KK, JH, SC. Contributed reagents/materials/analysis tools: KK, JH, SC. Wrote the paper: JP, JH, SC.

## Supporting information



Fig S1

Fig S2

Fig S3
