# Supplementary figures and images for: Daily injection of melatonin inhibits insulin resistance induced by chronic mealtime shift
Source: Physiol Rep. 2022 Mar 28;10(6):e15227. doi: 10.14814/phy2.15227 (PMC8958345; doi:10.14814/phy2.15227)

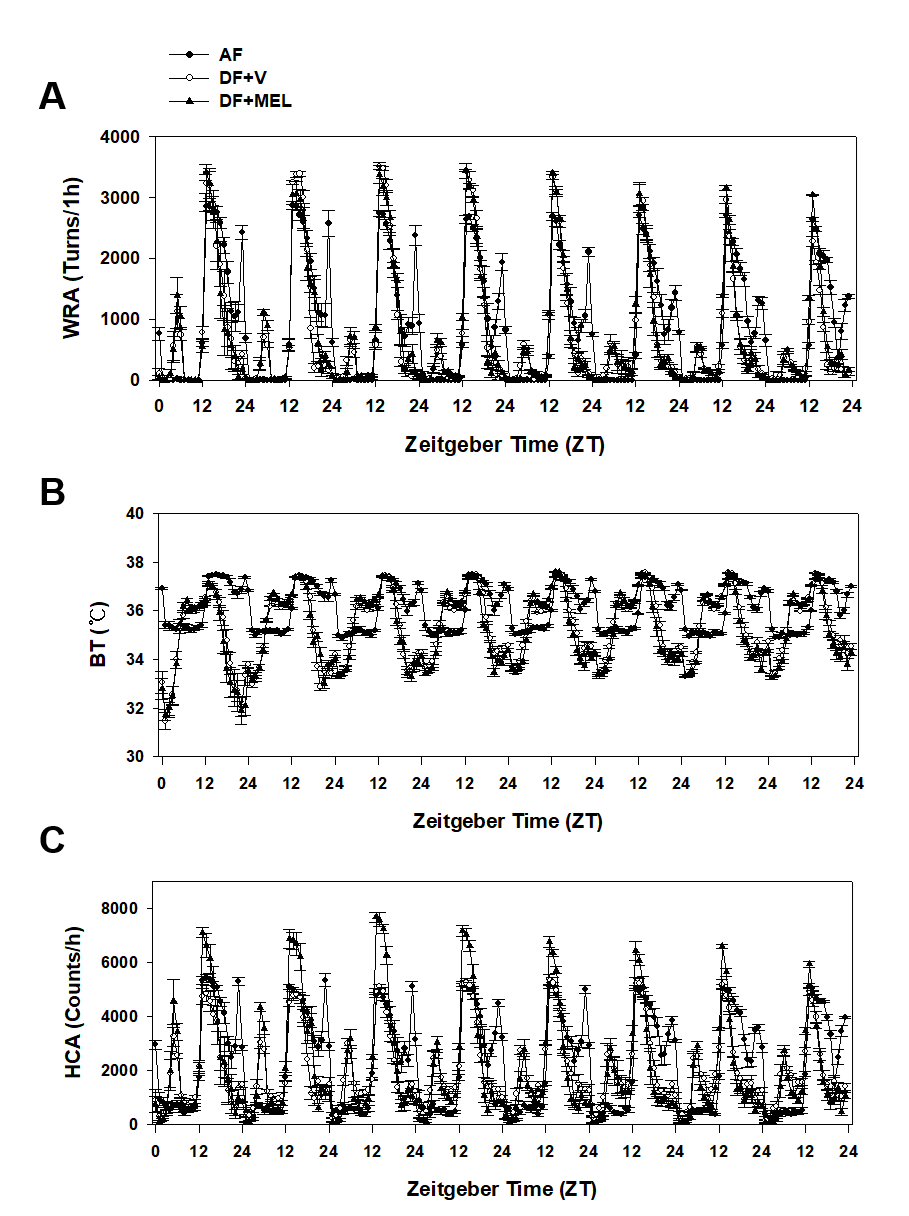

Supplement: Supplementary file 1 — Fig S1 [file PHY2-10-e15227-s003.tif]

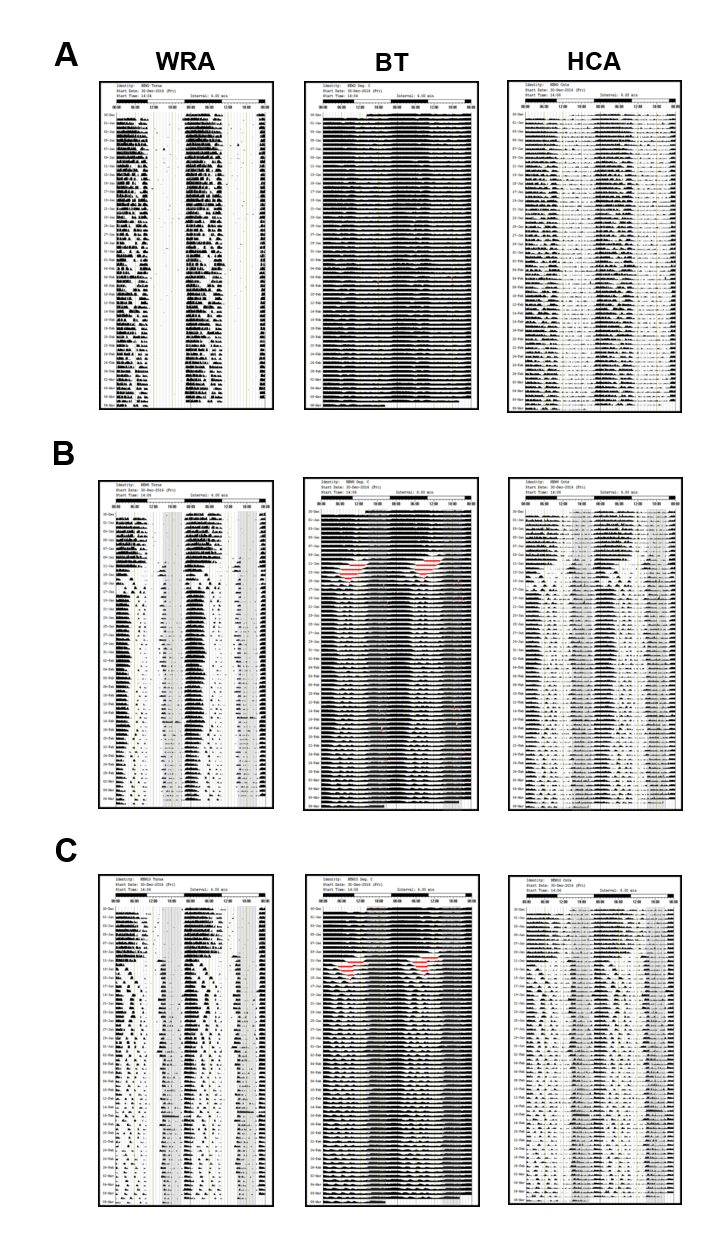

Supplement: Supplementary file 2 — Fig S2 [file PHY2-10-e15227-s002.tif]

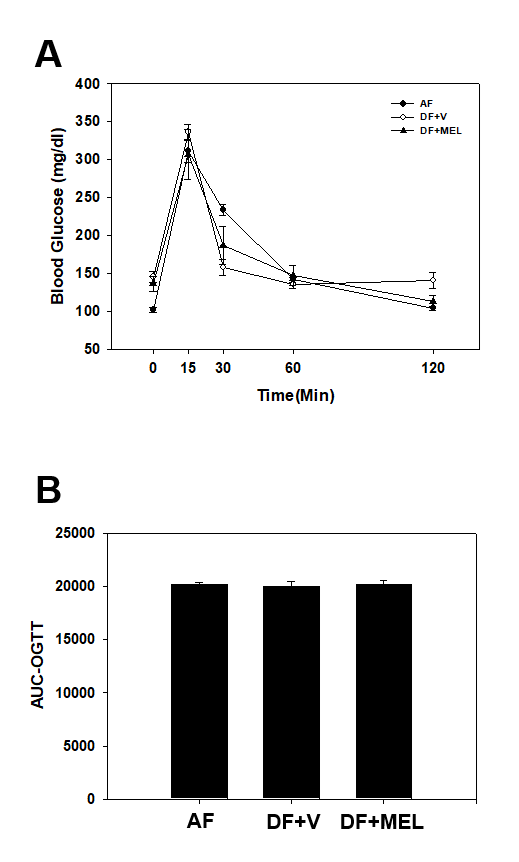

Supplement: Supplementary file 3 — Fig S3 [file PHY2-10-e15227-s001.tif]
